# Paralog-aware assembly and filtering strategies reveal minimal nucleotide variation on the macro germline-restricted chromosome of the zebra finch

**DOI:** 10.1038/s41437-026-00830-z

**Published:** 2026-03-16

**Authors:** Augustin Chen, Francisco J. Ruiz-Ruano, Orlando Contreras-López, Simone Fouché, Alexander Suh, Yifan Pei

**Affiliations:** 1 Department of Organismal Biology—Systematic Biology, Science for Life Laboratory, Uppsala University, Uppsala, Sweden; 2 Stochastic Models for the Inference of Life Evolution (SMILE), CIRB, Collège de France, Université PSL, CNRS, INSERM, Paris, France; 3 Institut du Cerveau-Paris Brain Institute-ICM, Sorbonne Université, Inserm, CNRS, Hôpital de la Pitié Salpêtrière, Paris, France; 4 School of Biological Sciences—Organisms and the Environment, University of East Anglia, Norwich Research Park, Norwich, UK; 5 Centre for Molecular Biodiversity Research, Leibniz Institute for the Analysis of Biodiversity Change, Museum Koenig Bonn, Bonn, Germany; 6 Bonn Institute for Organismal Biology (BIOB)—Animal Biodiversity, University of Bonn, Bonn, Germany; 7 Science for Life Laboratory, Department of Gene Technology, KTH Royal Institute of Technology, Solna, Sweden

**Keywords:** Genetic variation, DNA sequencing, Haplotypes, Bioinformatics

## Abstract

The germline-restricted chromosome (GRC) of passerines is a remarkable tissue-specific chromosome that accumulated paralogs of genes from the regular “A chromosomes” over millions of years, often amplified into dozens of gene copies. In addition to its repetitive content, typically uniparental inheritance, and lack of recombination, the GRC resembles non-recombining sex chromosomes and some B chromosomes, for all of which assembly and single-nucleotide polymorphisms (SNPs) calling are difficult. Here, we first show that much of the Australian zebra finch macro-GRC can be assembled using accurate long reads. We then describe a paralog-aware Snakemake pipeline, ParaVar, to map short reads from the GRC to retrieve GRC regions suitable for haplotype-based analysis. ParaVar reliably calls hundreds of SNPs across the GRC, thereby providing an estimate of nucleotide diversity on the highly repetitive zebra finch macro-GRC. Our results show significantly lower nucleotide diversity (20- to 50-fold lower) on the GRC compared to the mitogenome and autosomes, and a strong phylogenetic discordance between the GRC and the mitochondrial genome. Beyond the contribution of background selection, our results suggest that a single GRC haplotype recently spread through the populations while jumping across matrilines via occasional paternal inheritance. We anticipate that our paralog-aware pipeline will be useful for SNP calling and population genetics analyses of repetitive GRCs, sex chromosomes, and B chromosomes.

## Introduction

The passerine germline-restricted chromosome (GRC) is a peculiar chromosome characterised by its programmed elimination from somatic tissues during early development and from mature sperm during spermatogenesis (Borodin et al. 2022; Ruiz-Ruano et al. 2025). Following its typical matrilineal inheritance, the GRC is observed as a single copy in male spermatogonia, but as two copies—presumably after duplication of itself—in female primary oocytes (Borodin et al. 2022). Long known as a genetic oddity of the zebra finch (Pigozzi and Solari 1998) and the Bengalese finch (del Priore and Pigozzi 2014), the GRC was recently shown to be present in ~6700 passerine species (Torgasheva et al. 2019; Borodin et al. 2022; Ruiz-Ruano et al. 2025), which represent two-thirds of all bird species. Cytogenetic analyses across 27 songbird species revealed remarkable variations in GRC size (Borodin et al. 2022), with assemblies ranging from micro-GRCs over meso-GRCs to macro-GRCs (Schlebusch et al. 2023; Mueller et al. 2023; Ruiz-Ruano et al. 2025). Remarkably large size differences can exist even between GRCs of closely related species. For example, *Lonchura malacca* has a micro-GRC while *L. castaneothorax* possesses a macro-GRC, despite being separated only ~2 million years ago (Sotelo-Muñoz et al. 2022). In general, passerine GRC size changes across the phylogeny suggest fast rates with independent events of massive additions and deletions (Torgasheva et al. 2019).

Recent studies have focused on characterising and comparing the content of passerine GRCs across species, and inferring their evolutionary origin. Passerine GRCs have accumulated paralogs of genes from the rest of the nuclear genome (A chromosomes) over millions of years (Kinsella et al. 2019; Schlebusch et al. 2023; Mueller et al. 2023), with the most recent additions dating back to less than a million year ago, based on sequence divergence from their A-chromosomal paralogs (Kinsella et al. 2019). Some GRC paralogs are nearly identical to their A-chromosomal paralogs (Asalone et al. 2021) and are often highly amplified, such as a fragment of the *dph6* gene in the zebra finch, which has over 308 copies (Kinsella et al. 2019). These characteristics, together with typical matrilineal inheritance, strongly resemble those of supernumerary B chromosomes (Houben et al. 2025). Unlike B chromosomes that are absent from certain individuals and vary in copy number between individuals from the same species, the GRC is present in all studied individuals—with typically a single copy in males and two copies in females (Borodin et al. 2022). As the second copy in females most likely arises from self-duplication, the GRC might evolve as a single non-recombining haplotype (Pei et al. 2022a), with reduced effective population size (¼ that of autosomes), like the non-recombining female-specific W chromosome. It is possible that the GRC originated as a B chromosome that later acquired essential germline functions (Ferree et al. 2024; Houben et al. 2025), and it is likely to be involved in adaptation with the largely co-inherited W chromosome and mitogenome. Because genomic regions such as GRCs, B chromosomes, sex chromosomes, and pericentromeric regions tend to be highly repetitive (Camacho 2005; Carey et al. 2022; Jaegle et al. 2023), they all pose major challenges for genome assembly and population genetic analysis.

Despite continued interest in understanding the genetic content, variation, and evolution of the passerine GRC across species, little is known about its evolution and variation within a species. To our knowledge, the only haplotype-based between-population genomic study of a passerine GRC was done on the Australian zebra finch (*Taeniopygia guttata castanotis*; Pei et al. 2022a), which has a macro-GRC estimated to be around 167.3 Mb in size (Kinsella et al. 2019). Attempts to assemble this highly repetitive macro-GRC have been particularly challenging. The first GRC assembly (Kinsella et al. 2019) consisted of 36 high-confidence scaffolds with a total length of 1.24 Mb. These GRC scaffolds were extracted from a linked-read draft testis assembly using GRC SNVs and manual curation. Later, Asalone et al. (2021) identified 720 additional GRC contigs from the same draft testis assembly using a coverage-based analysis, extending the GRC assembly to 4.79 Mb. While this draft GRC assembly enabled assigning sequences to the GRC with high confidence, it is not necessarily suited for assessing intraspecific genetic variation. We hereafter define regions where intraspecific genetic diversity can be assessed as “scorable” and the extent to which a region is “scorable” as “scorability”. The first set of scorable GRC regions was identified by Pei et al. (2022a) and had a total length of about 100 kb. Using a linked-read testis library consisting of 150-bp read pairs with barcodes for haplotype information, and focusing on low-copy GRC regions highly diverged from their A-chromosomal paralogs, GRC sequences were assembled via multiple rounds of mapping, selection, and assembly of reads sharing the same linked-read barcode. This set of scorable GRC regions suggested extremely low nucleotide diversity and incongruent phylogenetic trees between the GRC and the mitochondrial genome. However, this represents less than 1% of the estimated size of the zebra finch macro-GRC (167.3 Mb; Kinsella et al. 2019).

Generating a germline reference genome with most of the GRC assembled, along with a high-quality A-chromosomal assembly, should allow us to quantify and identify all scorable regions of the GRC. Accurate Pacific Biosciences HiFi long reads (read length up to 25 kb) proved their performance in assembling multi-copy near-identical repeats in the human genome (Nurk et al. 2022). Recently, accurate long reads have also been used to assemble the 6.5–9.7 Mb blue tit (*Cyanistes caeruleus*) micro-GRC (Mueller et al. 2023). Very recent preprints suggest that long-read GRC assemblies from other species will be published soon (Fang and Edwards 2025; Ruiz-Ruano et al. 2025).

We generated the first passerine macro-GRC assembly using accurate long reads, showing that about half of the highly repetitive Australian zebra finch macro-GRC can be confidently assembled and identified. Due to the high sequence similarity between A-chromosomal and GRC paralogs (Kinsella et al. 2019; Asalone et al. 2021; Pei et al. 2022a), we expect mismappings of A-chromosomal reads to GRC contigs, as well as between highly similar GRC paralogs, which could lead to false-positive GRC SNPs caused by between-paralog differences. Therefore, to reliably call SNPs on the GRC, it is necessary to exclude sites affected by mismapping. We first identified and defined three types of mismapping: (1) mismapping of A-chromosomal paralogs on GRC contigs (AChr-mm), (2) mismapping of unassembled GRC paralogs to GRC contigs (GRC-mm), and (3) ambiguous mapping of GRC reads due to GRC paralogs present in multiple copies with highly similar sequences (GRC-amb). We then designed ParaVar, a paralog-aware pipeline to minimise each type of mismapping and reliably filter out regions with AChr-mm, GRC-mm, and GRC-amb. As a result, we are able to identify scorable regions across the highly repetitive GRC assembly and call high-confidence GRC SNPs. We then use these SNPs to measure between-population genetic variation of the GRC across nine individuals from six different Australian zebra finch populations. Lastly, we compare our estimates of nucleotide diversity and GRC phylogeny to the previously studied ~100 kb of scorable GRC regions (Pei et al. 2022a).

## Materials and methods

### Animals and tissue collection

The two male zebra finches (SR06033 and SR18080) used to build the HiFi testis assembly (HiFi_tes) are closely related to individual SR00100, used as a draft germline genome assembly by Kinsella et al. (2019) (see pedigree in Table S1). All three individuals are from matriline A and belong to the domesticated “Seewiesen” population at the Max Planck Institute for Biological Intelligence, Seewiesen, Germany. This population was derived from one at the University of Sheffield (for details, see population #18 of Forstmeier et al. 2007). Male SR06033 died at the age of 1874 days in 2011 due to natural causes, and we stored its body at −20 °C until 2020 before dissection. We sacrificed and immediately dissected male SR18080 at 562 days of age. It is unclear whether these males were sexually active, but at dissection, their testes were of normal size, measuring approximately 3–4 mm in length. For each male, we dissected testes and a sample of liver, and flash-froze them before preparing the sequencing library. We conducted this study under the permit no. 311.4-si, Landratsamt Starnberg, Germany. We summarised details about the other birds from a publicly available dataset used in this study in Table S2.

### Tissue collection, DNA extraction, library preparation, and sequencing

We generated the HiFi_tes assembly by sequencing one of the testes from male SR18080 to generate a highly accurate long-read library via PacBio HiFi technology and one of the testes from male SR06033 to generate a Dovetail Omni-C library with information from chromatin conformation capture (Hi-C) for phasing (see details in Table S2).

To generate the PacBio HiFi library, we extracted high molecular weight (HMW) DNA from snap-frozen testis tissue using the Nanobind Tissue Big DNA Kit 1.0 (Circulomics) following the standard dounce homogeniser protocol described in the Nanobind HMW Tissue DNA Kit Handbook 0.16 d (4/19) and eluted in a 200 µl kit EB. Then, we constructed a PacBio SMRTbell™ library using the SMRTbell™ Express Template Prep Kit 2.0 & SMRTbell™ Enzyme Cleanup Kit following the instructions described in “Procedure & Checklist—Preparing HiFi SMRTbell Libraries using SMRTbell Express Template Prep Kit 2.0” (PN 101-853-100 Version 03 (January 2020)). In brief, we sheared 15 µg of genomic DNA into ~20 kb fragments using a Megaruptor 3 (Diagenode). After removal of ssDNA overhangs, DNA damage repair and end-repair/A-tailing, we ligated DNA fragments to hairpin adaptors, thereby generating the SMRTbell™ library for circular consensus sequencing. We then treated the library with nuclease treatment and selected fragments by size using the SageELF system (SageScience), and the resulting fraction with suitable fragment length was sent for sequencing. We performed primer annealing and polymerase binding using the Sequel II binding kit 2.0 and Sequencing Primer v2. We sequenced a total of 3 Sequel SMRT Cells 8 M v3 on the Sequel II system using Sequel II Sequencing Plate 2.0 with an on-plate loading concentration of 90 pM, movie time of 24 h, and pre-extension time of 2 h.

To generate the Hi-C library, we performed chromatin conformation capture on snap-frozen testis tissue using the Dovetail Omni-C Kit (Cat. nos. 21005, 21006, 21007, 21003) following the Omni-CTM Proximity Ligation Assay Non-mammalian Samples Protocol version 1.0, B. Animal Tissues (other than insects and marine invertebrates). Briefly, we used 17 mg of flash-frozen powdered tissue for crosslinking. We first digested the chromatin using DNase I in an in situ reaction. We then extracted the crosslinked genomic DNA and used it for proximity ligation. Next, we ligated spatially proximal digested DNA ends, enriched the ligated biotin-containing fragments, which we finally used to prepare an Illumina library. We finally ran the barcoded Hi-C library on an S4 flow cell of an Illumina NovaSeq 6000 machine with a run setup 151-8-8-151 XP. We generated around 180 million reads from our sample.

### The HiFi GRC assembly

To generate the HiFi_tes assembly, we first excluded HiFi reads ≤15 kb using SeqKit 2.0.0 (Shen et al. 2016). We then assembled the remaining HiFi reads together with the Hi-C data using hifiasm 0.15.5-r352 (Cheng et al. 2022). As the Australian zebra finch GRC is mainly a collection of paralogs of A-chromosomal sequences (Kinsella et al. 2019; Asalone et al. 2021; Pei et al. 2022a), assembling it can be thought of as effectively creating a third haplotype in addition to the diploid A chromosomes. To avoid discarding potential GRC contigs, we disabled the automatic purging of haplotig duplications by using the -l 0 from hifiasm while keeping other options set to default. This resulted in the HiFi_tes assembly, which contains contigs from both A chromosomes and the GRC.

Next, we labelled HiFi_tes contigs as either belonging to the GRC (HiFi_GRC) or the A-chromosomes (HiFi_Achr) via a conservative approach to include only high-confidence GRC sequences. For this, we first searched in HiFi_tes for hits to the previously published 115 high-confidence GRC genes (Kinsella et al. 2019) using the integrated BLASTN plugin of the assembly graph visualiser Bandage 0.8.1 (Wick et al. 2015). We considered all contigs within connected components containing BLASTN hits as potentially belonging to the GRC. Among the selected contigs, we identified A-chromosomal contigs by aligning them against the high-quality Australian zebra finch A-chromosomal reference genome from the Vertebrate Genomes Project (VGP; GCA_003957565.4; Rhie et al. 2021) using minimap2 2.30 (Li 2018) with options -cx asm5, excluding contigs aligning at least 95% of their length from the same A chromosome. We labelled the resulting contigs as HiFi_GRC and the others as HiFi_Achr. We provide assembly statistics for HiFi_tes, HiFi_GRC, and HiFi_Achr generated with gfastats 1.3.6 (Formenti et al. 2022) in Table S3. To assess the completeness of HiFi_tes, we computed a dot plot alignment between HiFi_tes and the Vertebrate Genome Project (VGP) A-chromosomal reference assembly (GCA_003957565.4; Rhie et al. 2021) using D-GENIES 1.5.0 (Cabanettes and Klopp 2018). We then used the assignment file from D-GENIES to calculate the percentage of VGP assembly covered by HiFi_tes (Table S4) using the function bedtools coverage from the BEDTools suite 2.30.0 (Quinlan and Hall 2010). To assess the structure of the GRC contigs, we first sorted the GRC contigs based on their homology with the A chromosomes by aligning them against the VGP somatic assembly (GCA_003957565.4; Rhie et al. 2021) using D-GENIES. Thus, we obtained the GRC contigs sorted by A-chromosome number and concatenated to generate a dot plot with ModDotPlot 0.8.2 (Sweeten et al. 2024).

Finally, we annotated genes on the HiFi_GRC contigs. For this, we first collected 12 published zebra finch RNA-seq libraries from the Sequence Read Archive (SRA) from adult testis and ovaries from Biederman et al. (2018) (SRR6896648, SRR6896649), Yin et al. (2019) (SRR7031411), the VGP (SRR8551565, SRR8551567, SRR8695301), and Kinsella et al. (2019) (SRR9671219, SRR9671220). We then mapped these RNA-seq libraries against HiFi_GRC using TopHat2 2.1.1 (Kim et al. 2013). Finally, we used GeMoMa 1.9 (Keilwagen et al. 2019) with default parameters to annotate HiFi_GRC using the mapped RNA-seq libraries and the gene annotation GCF_003957565.2 of the VGP assembly (Rhie et al. 2021). As an estimation of the gene completeness, we extracted protein sequences from all the annotated genes with AGAT 1.3.0 (Dainat 2024). Then, we aligned these proteins against the NCBI NR protein database with DIAMOND 2.1.9.163 (Buchfink et al. 2015) with options blastp --very-sensitive -e 1e-6 --outfmt 6 qseqid sseqid pident length mismatch gapopen qstart qend sstart send evalue bitscore salltitles qcovhsp scovhsp. We obtained the coverage percentage for the first target protein for each gene.

### Whole genome sequencing data and mitogenome characterisation

For our between-population genetic analysis of the GRC, we used whole-genome sequencing (WGS) data from previously published studies (Kinsella et al. 2019; Pei et al. 2022a) to select mitochondrial haplotypes that span the broadest available range of the mitochondrial nucleotide diversity (Table S2 and Fig. S1B).

Briefly, we included nine male individuals representing nine matrilines or mitochondrial haplotypes from six different populations (six domesticated individuals, two recently wild-derived individuals, and one wild individual collected from Australia; Table S2). Each individual generated two DNA libraries: one from somatic tissue (liver or leg muscle) and one from testis (i.e., containing germline and soma). All libraries were sequenced on an Illumina HiSeq X or Illumina NovaSeq 6000 sequencing platform using paired-end short reads or linked reads (10X Chromium), except for the testis library of individual SR00100, which was sequenced twice to increase the total read depth (see details in Table S2). Note that individual SR00100 was also used to generate the previous GRC reference assembly by Kinsella et al. (2019) and is from the same matriline as the two individuals SR18080 and SR06033 that we used in our study to obtain our HiFi_tes (see pedigree chart in Table S1).

### Raw reads processing, key statistics extraction, variant calling and quality control

We first trimmed all Illumina libraries using Trimmomatic 0.39 (Bolger et al. 2014) with the options ILLUMINACLIP:TruSeq3-PE-2.fa:2:30:10 LEADING:3 TRAILING:3 SLIDINGWINDOW:4:20 MINLEN:100 as in Kinsella et al. (2019). We then mapped trimmed somatic and testis libraries using BWA-MEM2 2.2.1 (Vasimuddin et al. 2019) against the VGP assembly (GCA_003957565.4; Rhie et al. 2021) plus the aforementioned HiFi_GRC contigs, which together make the reference genome VGP+HiFi_GRC. Because HiFi_Achr contains a mix of phased and unphased haplotypes, we included the VGP assembly (excluding the W chromosome and mitochondrial genome) instead to facilitate mapping.

To identify regions on the GRC that are suitable for haplotype analysis, we focused on the following statistics for each GRC contig: the read depth per individual per site (DP), the mapping quality scores per site (i.e., mean mapping quality score, MQ), as well as the fraction of reads with mapping quality score equal to 0, MQ0F). We called genotypes while extracting the aforementioned statistics on the GRC for downstream sites selection, population genetic and phylogenetic analyses. To retrieve these statistics, we fed all BAM files to bcftools mpileup 1.21 (Danecek et al. 2021) while disabling the filter on read depth (--max-depth 2147483647) and requiring computing the read depth per individual per site (-a FORMAT/DP). We then piped the output of bcftools mpileup to bcftools call, which we ran using the --multiallelic-caller option. Conveniently, bcftools outputs genotype, MQ, MQ0F and DP for both variant and invariant sites by default in a single VCF output. We used VCFtools --geno-depth 0.1.17 (Danecek et al. 2011) to extract DP from the VCF file.

We kept only sites with genotype missing at most in one individual using VCFtools using --max-missing 0.8. Additionally, for variant sites, we kept only SNPs with a minimum Phred-scaled variant quality score of 30.

Next, for each sample, we used samtools depth 1.21 to retrieve read depth per site across A chromosomes, then calculated median read depth from 1,000,000 sites uniformly sampled for library size correction.

### Site filtering for A-chromosomal mismapping

As we expect reads from A-chromosomal paralogs to mismap on the GRC (AChr-mm), we designed the somatic mismapping (SM) statistic, which corresponds to the average fraction of reads from somatic libraries mismapped on the GRC per site. SM is an estimator of the average fraction of A-chromosomal reads from testis libraries that mismap on the GRC. We defined SM as follows:

$$SM\,=\frac{1}{9}\cdot \mathop{\sum }\limits_{i=1}^{n=9}\frac{D{P}_{som{a}_{i}}}{D{P}_{testi{s}_{i}}}\cdot \frac{L{S}_{A-chr\,of\,testi{s}_{i}}}{L{S}_{A-chr\,of\,som{a}_{i}}}$$

where $D{P}_{som{a}_{i}}$ and $D{P}_{testi{s}_{i}}$ are the somatic and testis read depth at a given site, and $L{S}_{A-chr\,of\,som{a}_{i}}$ and $L{S}_{A-chr\,of\,testi{s}_{i}}$ are the median read depth on A-chromosomes for a given somatic and testis sample, to account for overall library size differences. Hence, $S{M}_{i}=\frac{D{P}_{som{a}_{i}}}{D{P}_{testi{s}_{i}}}\cdot \frac{L{S}_{{\rm{A}}-chr\,of\,testi{s}_{i}}}{L{S}_{A-chr\,of\,som{a}_{i}}}$ corresponds to the read depth ratio of the matched somatic and testis libraries *i* normalised by their difference in library size. SM is simply the average $S{M}_{i}$ across all nine individuals. To test the effect of AChr-mm on population genetic and phylogenetic analyses, we set different thresholds by keeping only sites with 0%, ≤1%, ≤10%, and ≤30% of SM. We provide an overview of the filtering steps in Fig. 2 and a complete record, including filtering parameters and the resulting number of sites in Table S5.

### Site filtering for GRC-paralog mismapping

The zebra finch macro-GRC is known to be highly repetitive; we expect two GRC mismapping issues from the highly repetitive chromosome with many (near-)identical repeats, particularly for short-read libraries: (1) mismapping from unassembled GRC-paralogs (GRC-mm) and (2) ambiguous mapping due to the high identity level between assembled GRC-paralogs (GRC-amb). Therefore, we applied two types of filtering to account for these issues.

To identify GRC regions with GRC-mm, we applied a filter on read depth. To take account of differences in library size, as well as variation in the amount of GRC reads due to differences in males’ sexual activity level during sample collection, we incorporated an “expected read depth interval” of a minimum of 1 and a maximum of twice the median read depth across regions known with high confidence to be single-copy. To do so, we first located a collection of high-confidence single-copy regions by finding previously published GRC sequences present in single-copy with high confidence (Pei 2022b) on our newly assembled GRC contigs, and only kept sites with SM = 0% (Fig. S2). Then, for each testis sample, we calculated the median read depth across these high-confidence single-copy regions and highlighted sites where read depth was equal or higher than 1 and equal or lower than twice the median calculated from single-copy regions. Finally, we considered GRC sites to have an acceptable level of GRC-amb if 5 or more testis samples had a read depth within their single-copy interval. Note that this interval can be simplified depending on prior knowledge of the copy number across the target genomic region. To filter out GRC-amb, we kept sites with a minimal MQ value of 30 and a maximum MQ0F value of 25%.

### Suitable GRC regions for haplotype analysis

To finally select all suitable sites on the GRC, we defined scorability as the number of sites that pass all our filters, including missingness, quality score for variant sites, AChr-mm, GRC-mm and GRC-amb over the total number of sites in a given interval. We calculated scorability in 1-kb non-overlapping windows on HiFi_GRC and kept only those where ≥50% of sites were scorable for nucleotide diversity and phylogenetic analyses. We plotted scorability per 1-kb non-overlapping window using the R package RIdeogram 0.2.2 (Hao et al. 2020) and performed visual inspection of read alignment using the Integrative Genomics Viewer (IGV) 2.16.2 (Robinson et al. 2011).

### Sensitivity analysis of the GRC SNP calling pipeline

We benchmarked our pipeline using as positive controls 47 published high-confidence SNPs found in single-copy regions of the GRC from the same birds used in our study (Pei 2022b, “Fig4b-cas12_GRCsinglecopy_snpsPlsgaps.csv”). These 47 SNPs were obtained from the ~100 kb single-copy regions of the GRC generated via targeted mapping and barcoded assembly of linked reads at testis-specific regions (https://github.com/fjruizruano/In_Silico_SeqCap_10xG; Pei et al. 2022a).

We searched these 47 SNPs with their 100 bp upstream and downstream flanking regions using BLAT 36 (Kent 2002) against VGP+HiFi_GRC. We then kept only SNPs with BLAT hits in GRC contigs. Next, for each of those remaining SNPs, we searched filtered variant-sites VCF files for a variant at the expected SNP location across all BLAT hits. If not found, we searched the unfiltered variant-sites VCF file and, if missing again, the invariant-sites VCF file. We provide coordinates of the recalled high-confidence SNPs on VGP+HiFi_GRC and detailed recall results in Table S6.

### Nucleotide diversity

We estimated nucleotide diversity (π) across GRC contigs using pixy 2.0.0.beta12 (Korunes and Samuk 2021). For GRC contigs, we calculated π per 1-kb non-overlapping window as well as per annotation feature. We obtained our estimates per annotation feature by computing the ratio of the number of pairwise differences (numerator) to the total number of pairwise comparisons (denominator) between all genotypes across the region of interest. Following pixy’s recommendations (Korunes and Samuk 2021), we did not include pairwise comparisons involving missing genotypes. We visualised π using functions from the R library tidyverse 2.0.0 (Wickham et al. 2019)

Unlike the GRC, where a somatic sample from the same individual is an ideal negative control sample because the haplotypes only differ by the presence or absence of the GRC, other cases, such as B chromosomes or W chromosomes, do not have such suitable negative controls. Therefore, we studied the effect of scorability and AChr-mm on downstream analyses. To compare the GRC with other genomic regions, as well as between GRC regions with different levels of scorability and SM (i.e., estimator of AChr-mm), we used unpaired t-tests, linear models, and linear mixed-effect models in R 4.1.3 (R Core Team 2021). Note that as window-based estimates of GRC π follow a Poisson distribution (see Results), we used the function t.test() from the R package stats to test for the significance and direction of differences between the GRC π and the published values of mitochondrial and autosomal π, while using medians to discuss the fold changes in π between these different genomic regions.

To test if different genomic annotations yield different π values for the GRC, we used the lmer() function from the lme4 1.1-27 R package (Bates et al. 2015). For this, we fitted π estimates across different annotation regions on the GRC with different SM tolerance levels as the response variable and fitted the annotation type as a factor variable with four levels (i.e., CDS as baseline, gene, intron with UTR, and unannotated) while controlling for the different SM tolerance levels as a covariate. We additionally included the contig ID as a random effect to control for pseudoreplication. To account for differences in region size (median = 252 bp, range from 1 to 23,217 bp), we weighted each estimate by the length of the corresponding region in kilobases.

To compare the π estimates between synonymous and nonsynonymous sites, we first identified synonymous and nonsynonymous sites in each annotated CDS region using bcftools csq with haplotype-aware calling disabled via --local-csq. We then used the t.test() function from the stats 4.1.3 R package to compare the π differences between the two groups, only using sites with SM = 0%.

To study the distribution of scorability and SM across different contigs of the GRC assembly, we used two mixed-effect models, with either the scorability per 1-kb window or SM per 1-kb window as the response variable, respectively. For this, we used the lmer() function from the lme4 R package. For both models, we included the intercept and the contig ID of the window as a random effect to control for pseudoreplication. Next, we studied the effects of scorability and SM on π estimates on the GRC using a mixed-effect model. For this, we included π estimate per 1-kb window as response variable and contig ID as a random effect to control for pseudoreplication. For fixed effects, we included scorability and SM as two covariates to compare the effects of scorability and SM. For all models, all covariates and response variables are Z-transformed to calculate standardised effect sizes. *p* values were obtained by comparing the model with and without the focal fixed or random effect using the function anova() from the R package stats.

### Phylogenetic analyses

We inferred the GRC phylogenetic tree using RAxML 8.2.12 (Stamatakis 2014). To reduce computation time, we decided to only use variant sites. Our first step was to transform filtered variant-sites VCF files into a relaxed version of the PHYLogeny Inference Package (PHYLIP) format using vcf2phylip 2.0 (Ortiz 2019). We used the resulting PHYLIP matrices as input for raxmlHPC-PTHREADS_AVX2 to build the trees assuming a general time-reversible model and a discrete gamma distribution of rate heterogeneity via -m GTRGAMMA. We conducted a rapid bootstrap analysis and search for the best-scoring Maximum Likelihood (ML) tree in a single run using the -f a algorithm. We used -N autoMRE to automatically determine the sufficient number of bootstrap replicates, which uses an empirically determined bootstopping cutoff of 0.03 (Pattengale et al. 2010).

To select the set of GRC variants to use for the co-phylogenetic analysis, we compared the topology of phylogenetic trees inferred from each set (obtained using different somatic mismapping thresholds: 0%, ≤1%, ≤10%, ≤30%) using SplitsTree4 4.18.1 (Huson and Bryant 2006) and its SuperNetwork plugin (Huson et al. 2004). We selected the largest set that does not change the topology of the tree inferred using the set of variants with only 0% of SM and found the set with ≤ 10% of SM to be optimal (Fig. S3). We midpoint-rooted the selected GRC tree and compared it pairwisely with the published mitochondrial tree “fig4_mt.raxml.support” (Pei 2022b) using phytools 2.0-3 (Revell 2012).

## Results

### The germline-restricted chromosome assembly

**Fig. 1:**
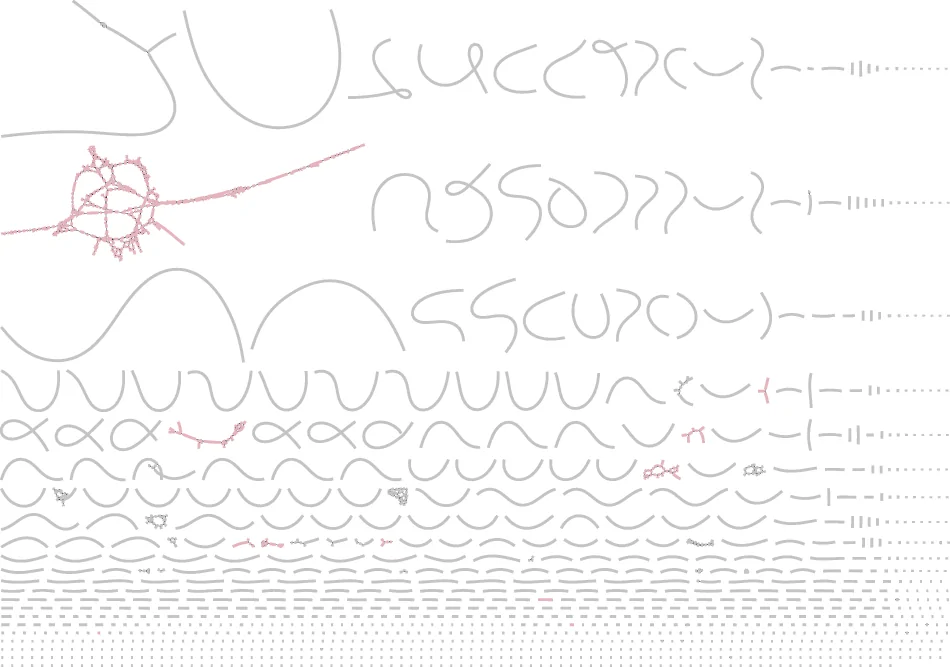
Assembly graph of the reference testis assembly (HiFi_tes) generated with Hifiasm using HiFi and Hi-C libraries. We selected GRC contigs (here in pink) as those within connected components containing high-confidence zebra finch GRC genes reported by Kinsella et al. (2019), after excluding contigs assigned to an A chromosome of the VGP somatic assembly.

The zebra finch testis assembly generated from accurate long reads (HiFi_tes; Fig. 1) is composed of 1941 contigs for a total length of 1.77 Gb (Table S3) and was assessed as near complete for A-chromosomes as it covers 99.2% (Table S4 and Data S1) of the high-quality somatic reference genome from the Vertebrate Genome Project (VGP). Contig length of HiFi_tes ranged from 15.25 kb to 39.39 Mb, with an N50 of 6.21 Mb (Table S3). Within this assembly, we identified 692 contigs to be associated with the germline-restricted chromosome (GRC) using a conservative approach via sequence homology to previously published high-confidence GRC genes (Kinsella et al. 2019; Fig. 1). This sums to a total length of 90.39 Mb, which represents 54.03% (Fig. S1A and Table S5) of the estimated 167.3 Mb GRC size (Kinsella et al. 2019). On the assembly graph, GRC contigs (HiFi_GRC) form tangled connected components, which is a hallmark of unresolved repeats, as contigs can be connected via multiple, equally possible paths (Fig. 1).

### Site-based filtering pipeline, the scorable regions, and variant sites on the GRC

**Fig. 2:**
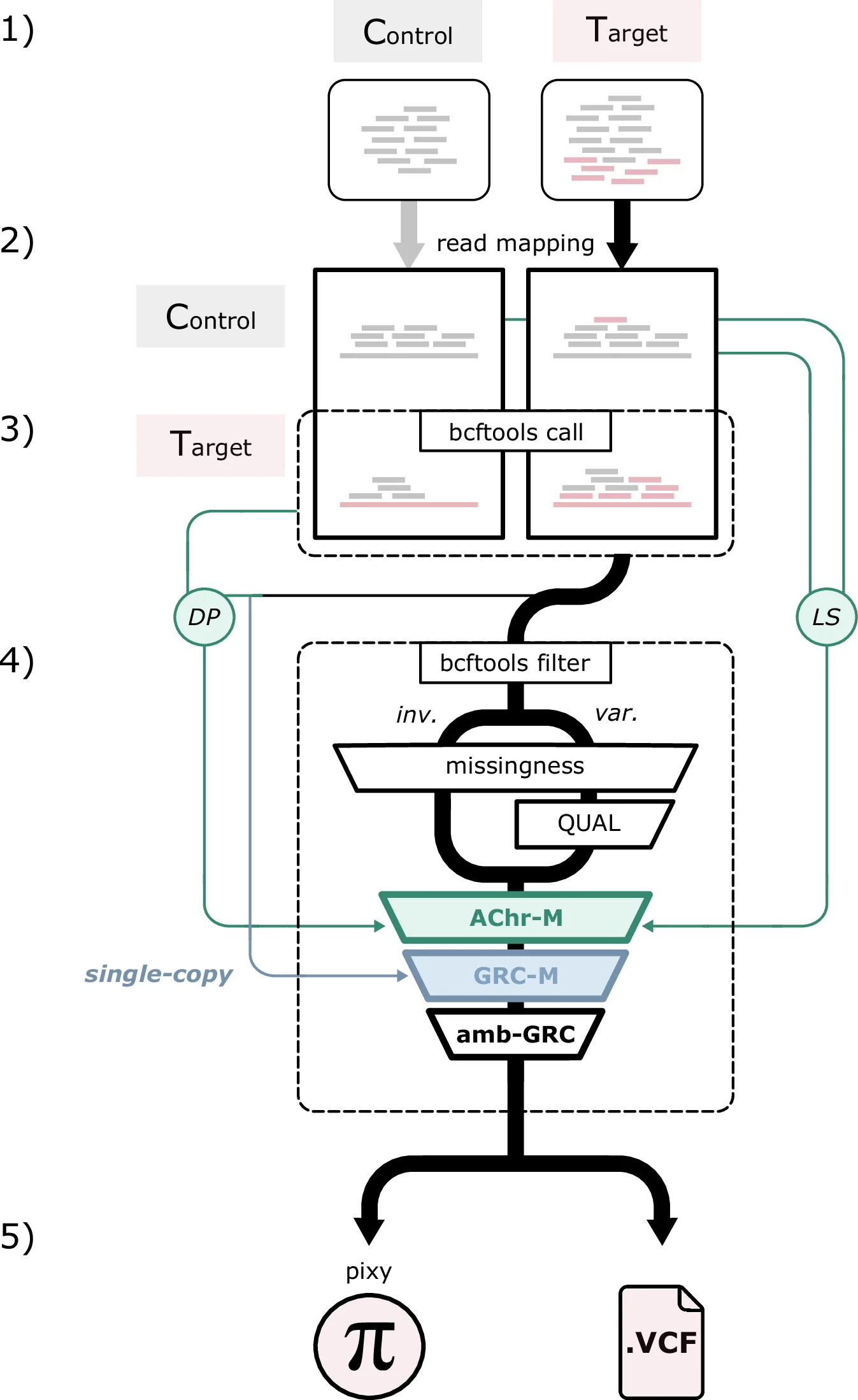
Paralog-aware pipeline to call SNPs from tissue-specific genomic regions. A simplified diagram of the paralog-aware pipeline ParaVar implemented in Snakemake 8.30.0 (Mölder et al. 2021) and used in this study. In step (1), from each individual zebra finch, a control (somatic) and a target (testis) Illumina libraries were obtained and trimmed. In step (2), trimmed reads were mapped to the reference assembly, which includes control (VGP) + target (HiFi_GRC) regions using, e.g., BWA-MEM2. Next, in step (3), calling of both variant and invariant sites is performed, on the target region (HiFi_GRC) specifically using bcftools call. Here, the read depth per sample per site (DP) and mapping quality statistics (MQ, MQ0F) were retrieved for filtering. In parallel, read depth information from the control (A-chromosomal) regions was retrieved and used to estimate library sizes (LS). Then, in step (4), were filtered out sites exhibiting A-chromosomal mismapping (AChr-mm) (in green), mismapping of unassembled GRC-paralogs on the target GRC contigs (GRC-mm) (shown in blue), as well as sites enriched in ambiguous multimapping reads (GRC-amb). (5) Finally, the list of scorable GRC sites is retrieved and used to calculate missingness-aware estimates of π with pixy.

Because of the high sequence similarity between GRC genes and their A-chromosomal paralogs, we expected mismapping of A-chromosomal reads to GRC contigs (AChr-mm), which could lead to the erroneous calling of GRC SNPs actually corresponding to differences between A-chromosomal and GRC paralogs. Similarly, we expected the high similarity between GRC-paralogs (Kinsella et al. 2019; Asalone et al. 2021; Pei et al. 2022a) to result in ambiguous mapping of GRC reads with matches to multiple locations with equal likelihood (GRC-amb; Fig. S4). Moreover, as only about half of the zebra finch macro-GRC could be assembled, we expect the mismapping of reads from unassembled GRC regions (GRC-mm), which could lead to false positive GRC SNP calls corresponding to differences between assembled and unassembled GRC paralogs (Fig. S4). Therefore, we designed the “somatic mismapping” (SM) statistic, which quantifies the average fraction of reads from somatic libraries mismapped on the GRC per site, and serves as a proxy for AChr-mm. We also designed a bespoke filter on read depth to exclude regions exhibiting GRC-mm (Fig. 2). For the multimapping issue caused by GRC-amb, we filtered on mapping quality scores. We implemented those filters as part of a “paralog-aware” reproducible Snakemake pipeline, ParaVar, designed to call SNPs from tissue-specific genomic regions such as the GRC (Fig. 2). The pipeline requires (1) libraries that contain reads from the tissue-specific genomic region of interest, (2) matched control libraries that do not contain the genomic region of interest, and (3) a reference genome with sequences from both the region of interest and the control region annotated as such (Fig. 2).

**Fig. 3:**
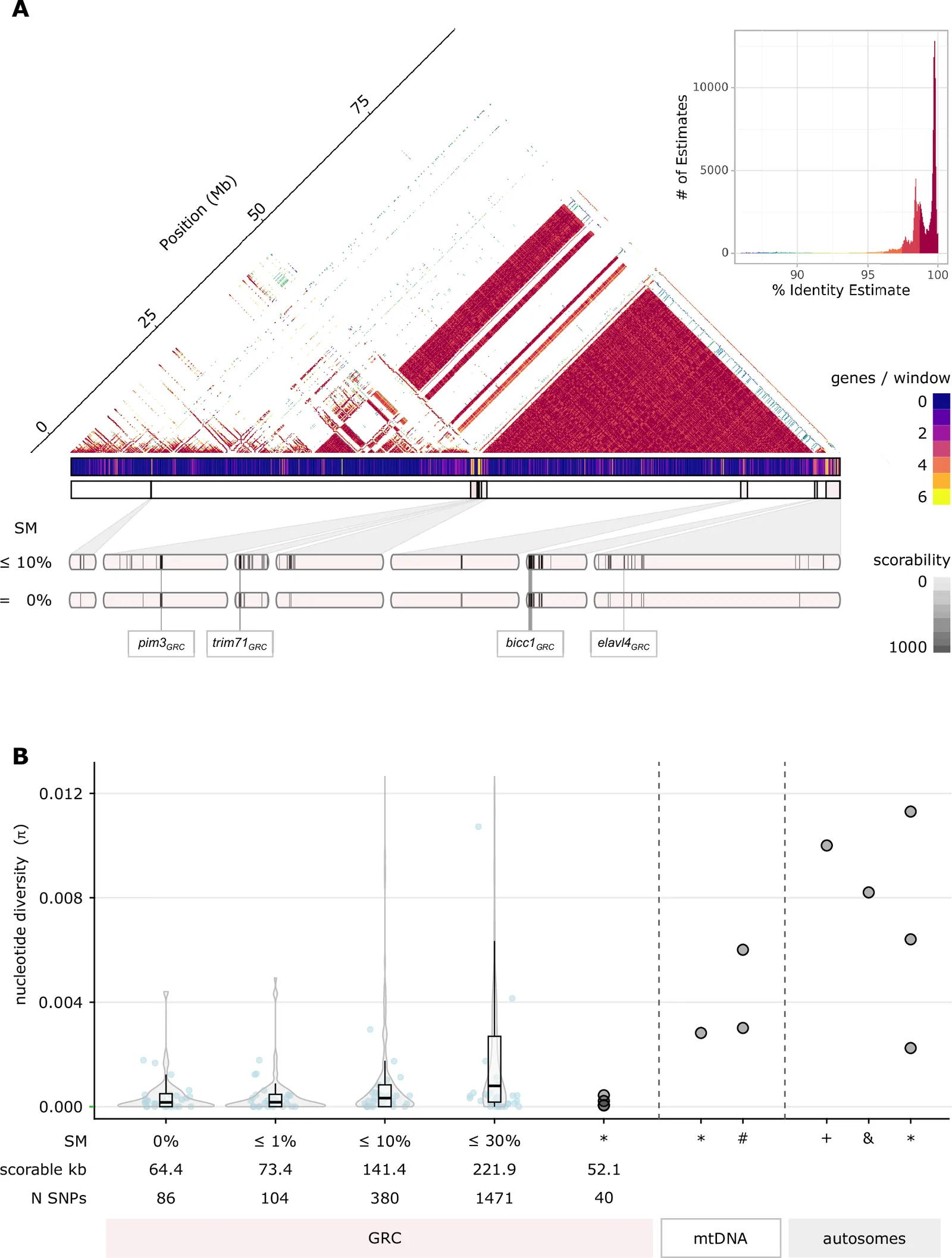
Overall structure and scorable regions of the GRC, and median GRC nucleotide diversity (π) estimates. **A** Dot plot showing sequence similarity along the 692 concatenated GRC contigs sorted by their homology to A-chromosomes. The histogram represents the number of pairs of 90-kb windows within a given identity interval, with low identity pairs (near 86%) shown in light blue and high identity pairs (near 100%) shown in dark red. The first ~48 Mb of the GRC corresponds to a repetitive region with homology to a region on chromosome 1 longer than 1 Mbp, containing the *gbe1*, *robo1*, and *robo2* genes, for example. The region from ~48 to ~87 Mb contains a short repeat of ~20 kb homologous with part of the *dph6* gene on chromosome 5. These two repetitive regions span the majority of the assembled GRC. Below the dot plot is a track illustrating gene density along the GRC contigs, calculated as the number of gene annotations per 90-kb non-overlapping window. The values go from 0 to a maximum value capped at 6 genes per 90-kb window. Just below, we indicated the positions of the seven GRC contigs with scorable windows and displayed the scorability values in a zoomed view, with light grey indicating low scorability and dark grey indicating high scorability. Additionally, we include the annotation of four GRC genes in the scorable GRC contigs. Please also see Fig. S5 for the distribution of gene density across different levels of completeness. **B** The distribution of GRC nucleotide diversity (π) estimates over 1-kb non-overlapping windows across different somatic mismapping (SM) thresholds is shown (violin and box plots), alongside previously reported values (grey dots) on the GRC, the mtDNA, and the autosomes. The GRC π in this study was estimated from nine zebra finch male individuals representing nine matrilines from six different populations. Previously reported π values are taken from * (Pei et al. 2022a, from the same nine birds as in this study), # (Newhouse and Balakrishnan 2015), + (Balakrishnan and Edwards 2009) and & (Singhal et al. 2015). Light blue dots correspond to 1-kb windows annotated as one of the GRC paralogs across which GRC π was estimated in Pei et al. (2022a): *elavl4*_*GRC*_*, bicc1*_*GRC*_, *pim3*_*GRC*_.

After applying the pipeline (Fig. 2), we obtained “scorable” regions from this highly repetitive chromosome (Fig. 3A and Table S5). During filtering steps, 98% of the assembled GRC contigs were identified to have GRC-amb, 36% showed significant GRC-mm and between 2 and 44% exhibited AChr-mm, value varying depending on the AChr-mm threshold. A fair fraction of sites displayed several mismapping issues (Table S7). These results are consistent with the fact that the majority of the assembled zebra finch macro-GRC consists of near-identical tandem repeats (Fig. 3A). Interestingly, the remaining “scorable” sites largely coincide with GRC regions of high gene density (Figs. 3A and S5).

We then studied the effect of SM on downstream results by allowing different SM fractions to be tolerated. This led to a total scorable length ranging from 64 to 438 kb, representing 0.04–0.26% (Fig. 3 and Table S5) of the estimated 167.3 Mb GRC size (Kinsella et al. 2019). We called 86, 104, 380, 1471, and 10,002 GRC SNPs from the scorable GRC regions, respectively, the number increasing with less strict SM thresholds: 0%, ≤1%, ≤10%, ≤30%, no SM filter (Fig. 3 and Table S5). Sensitivity analysis first located 33 out of 47 known GRC SNPs (Pei et al. 2022a; Pei 2022b) on our GRC assembly, the remaining SNPs being likely absent from our GRC assembly (Table S6). Initially, we successfully recalled all 33 GRC SNPs, but discarded 2 of them with low variant quality, and another 2 with high GRC-amb, which gives a final calling sensitivity of 87.88% (29 out of 33 SNPs; see detailed recall analysis in Table S6). Thereafter, we used our SNP set and the defined scorable GRC regions for downstream analyses.

### Nucleotide diversity across the germline-restricted chromosome assembly

GRC nucleotide diversity (π) was estimated over 1-kb non-overlapping windows, across different annotation features, and later separately for synonymous or nonsynonymous sites. All sites were used, regardless of whether they were synonymous or nonsynonymous. We found that, when allowing no AChr-mm, median estimates of π across the scorable GRC assembly are about 50 times lower than the autosomal estimate and 20 times lower than the mitochondrial estimate, the latter being marginally significant (i.e., keeping only sites with SM = 0% yields median_GRC_ = 0.00016, mean_GRC_ = 0.00039, median_auto_ = 0.0082, mean_auto_ = 0.0076, *t*_GRC vs. auto_ = −4.57, df_GRC vs. auto_ = 4.02, *P*_GRC vs. auto_ = 0.01; median_mito_ = 0.003, mean_mito_ = 0.0039, *t*_GRC vs. mito_ = −3.42, df_GRC vs mito_ = 2.02, *P*_GRC vs. mito_ = 0.075; Fig. 3B and Table S5). When partitioning the GRC into genes, coding sequences, introns and UTRs, as well as unannotated regions, we found no significant differences in π values among the different annotation features (0.1 < *Z* < 1.6, *P* = 0.1; Fig. S6). Inside coding regions, we found no significant difference between the π values estimated only from synonymous and nonsynonymous sites (keeping only sites with SM = 0%, mean_synonymous_ = 0.000094, mean_nonsynonymous_ = 0.00018, *t* = −0.792, df = 75.99, *P* = 0.43; Fig. S7).

Overall, we found that GRC π significantly increased with increasing SM (*r* = 0.62, *Z* = 16, *P* < 0.0001) but not with scorability (*r* = 0.06, *Z* = 1.8, *P* = 0.2) when disabling SM filtering. This was consistent with the expectation that AChr-mm contributes significantly to false positive SNPs. Additionally, we found that contig ID explained a significant amount of variation in SM (var_contig ID_ = 0.34, *P* < 0.0001), scorability (var_contig ID_ = 0.11, *P* < 0.0001), and π (var_contig ID_ = 0.17, *P* < 0.0001; also see Fig. S8). The effect of contig ID on SM reflects a non-random distribution of sequences with AChr-mm on the assembled GRC. However, the contig ID effect on π was gone when somatic mismapping was not allowed (var_contig ID_ = 0.12, *P* = 1; Fig. S9). Interestingly, using a relaxed somatic mismapping threshold (SM ≤ 10%), estimates of GRC π were still significantly lower than the autosomal π and marginally significantly lower than the mitochondrial π (across GRC windows, π ranges from 0 to 0.026; median_GRC_ = 0.00033, mean_GRC_ = 0.00096, *t*_GRC vs. auto_ = -4.19, df_GRC vs. auto_ = 4.10, *P*_GRC vs. auto_ = 0.013; *t*_GRC vs. mito_ = −2.83, df_GRC vs. mito_ = 2.12, *P*_GRC vs. mito_ = 0.098). We found high GRC π (i.e., in the same order of magnitude or larger than mitochondrial and autosomal estimates) in about 12% of the windows with SM ≤ 10% and 2% of those with SM = 0% (Fig. S10).

We also found that nucleotide diversity was somewhat significantly positively correlated with scorability across all GRC windows (*r* = 0.09, *Z* = 2.2, *P* = 0.03) after including contig ID as a random effect to control for pseudoreplication. However, when only keeping the windows with no somatic mismapping, the effect of scorability was no longer significant (*r* = −0.02, *Z* = −0.21, *P* = 0.53).

### Comparison of phylogenetic trees from the germline-restricted chromosome and the mitochondrial DNA

**Fig. 4:**
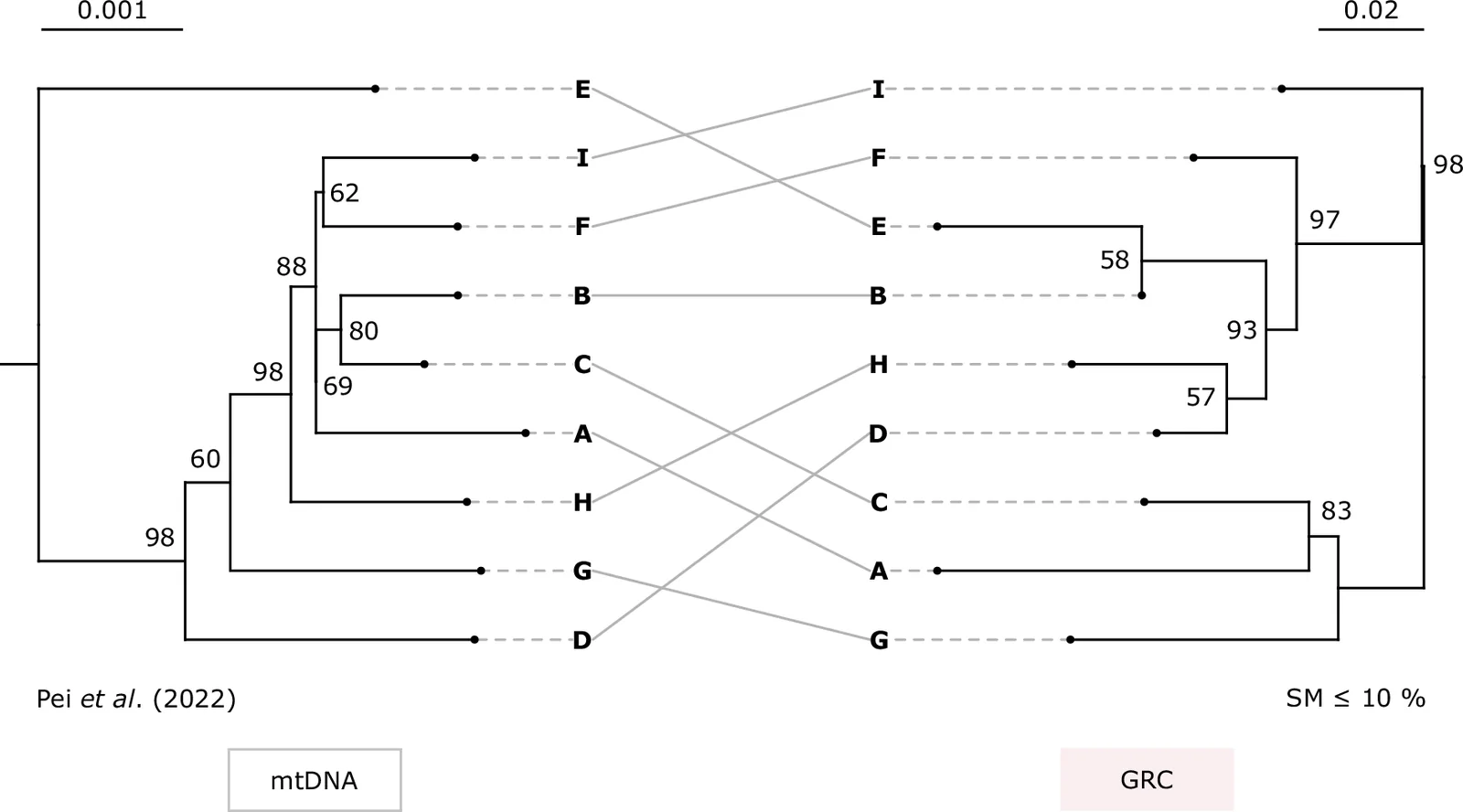
The GRC tree derived from 380 GRC SNPs is highly incongruent with the mitochondrial tree. The GRC tree (right) was built from 380 SNPs with ≤10% of somatic mismapping, while the mitochondrial tree (left) was obtained from Pei (2022b). The GRC tree is rooted on its midpoint, while the mitochondrial tree is rooted by the published mitochondrial sequence of the *T. g. guttata* datasets (Pei 2022b). For the cophylogenetic analysis, nodes were automatically rotated to maximise matching in vertical position. Because cophylo puts priority on symmetry, the two trees are on different scales (Revell 2012). The scale bars indicate the number of substitutions per site in the mitochondrial tree and the number of substitutions per variant site in the GRC tree. Numbers at nodes are bootstrap support values.

To study the level of co-inheritance between GRC and the mitochondria, we compared the topology of the phylogenetic trees derived from GRC SNPs and mitochondrial SNPs (Fig. 4). To minimise false-positive SNPs caused by AChr-mm, we only used GRC SNPs with acceptable somatic mismapping, i.e., SM ≤ 10%. As a result, a total of 380 SNPs were used to build the tree (Table S5). If the GRC were to follow a strict matrilineal inheritance, we would observe a completely matched topological relationship between the GRC and their mitochondrial haplotypes. However, comparison of this GRC tree to the mitochondrial tree reveals pervasive topological discordances. For example, the matrilines B, E, H, and D form a well-supported clade in the GRC tree but are scattered all around the mitochondrial tree (Fig. 4).

## Discussion

Our study shows that over half of the highly repetitive Australian zebra finch macro-GRC can be assembled by combining PacBio HiFi accurate long reads and chromatin conformation capture (Hi-C) for haplotype phasing (Fig. S1A, Tables S3, S4 and Data S1). A large proportion of the GRC assembly is composed of near-identical repeat arrays (Fig. 3A), against which uniquely mapping short reads is practically impossible. This was confirmed by our analysis, where 98% of the assembled GRC was filtered out as GRC-ambiguous mapping (GRC-amb; Fig. S11 and Table S7). This resulted in 222 kb of GRC assembly being assigned as scorable when some (≤30%) mismapping of A-chromosomal reads on the GRC (AChr-mm) was allowed (Table S5), which is double the ~100 kb of GRC studied previously (Pei et al. 2022a). Note that the resulting amount of scorable region largely depends on the level of repetitiveness of the target chromosome and read length from the study libraries. We believe that our pipeline could be even more effective with long-read-based sequencing data, and in less repetitive targets, such as micro-GRCs (Torgasheva et al. 2019; Schlebusch et al. 2023).

Window-based nucleotide diversity (π) analysis from all scorable GRC regions supports the previously reported (Pei et al. 2022a) lower π estimates on selected GRC regions compared to the autosomal, perhaps also mitochondrial sequences (Fig. 3B). In the absence of AChr-mm, we still found a few GRC windows exhibiting a high π (Fig. 3B, SM = 0%, outliers in GRC violin plot). Visual inspection of the alignment strongly indicated that most of them were caused by the mismapping of unassembled GRC paralogs (GRC-mm) as indicated by the presence of clusters of SNPs (within 100 bp) with a heterozygous genotype in most individuals, forming two distinct haplotypes (Table S8 and Figs. S12–S15). Those sites were missed by our GRC-mm filter, probably because their copy number is low. Our sliding window analysis of diversity allowed us to confidently identify these windows as outliers.

If the zebra finch GRC follows strict maternal inheritance, then GRC π is expected to be 25% of the autosomal π, and comparable to the W-chromosomal π. However, the observed ratio of GRC to autosomal π is 0.02; 12.5 fold lower than expected (Fig. 3B). Hence, additional mechanisms might be involved in further reducing π on the GRC. The zebra finch is a monogamous species with an equal sex ratio, a large effective population size (Ne), and without known population structure (Zann 1996). We note that the studied 9 zebra finch matrilines span the diversity of mitochondria in the wild (Pei et al. 2022a) and yield estimates of autosomal π comparable to the estimates from wild individuals (Balakrishnan and Edwards 2009; Singhal et al. 2015; Fig. 3B). These features make genetic drift unlikely to explain the observed low GRC diversity in our dataset. Recent selective sweeps and purifying selection were suggested to explain the reduced genetic diversities in the uniparentally inherited non-recombining Y-to-dot chromosome in flies *Drosophila pseudoobscura* (Larracuente and Clark 2014) and the Y chromosome in humans (Wilson Sayres et al. 2014). Previous gene-based analysis revealed that the zebra finch GRC contains at least 115 genes enriched in the function of female gonad development (Kinsella et al. 2019). Interestingly, some of these genes showed either signatures of long-term positive selection or long-term purifying selection (Kinsella et al. 2019; Biederman et al. 2018). Thus, genetic hitchhiking and background selection could contribute to the overall reduction of GRC diversity among zebra finches.

Multispecies cytogenetic data have shown that the passerine GRC is typically maternally inherited as it is expelled from the sperm during spermatogenesis (Borodin et al. 2022). However, in the zebra finch, the GRC can occasionally be paternally inherited, allowing GRC haplotypes to occasionally jump across matrilines and potential gene flux between GRC haplotypes either via gene conversion or recombination (Pei et al. 2022a). Our finding of major incongruence between the GRC and mitochondrial phylogenetic trees further supports the presence of historical GRC paternal inheritance (Fig. 4). Pei et al. (2022a) found repeatable differences in the fraction of GRC-carrying sperm between zebra finch males from two different families, suggesting that different GRC haplotypes may have different rates of paternal inheritance. We propose that (potentially selfish) GRC haplotypes with high rates of paternal inheritance might have contributed to the zebra finch’s low GRC nucleotide diversity by recently sweeping through the population, abruptly reducing Ne. This might also have allowed the fixation of copy number variants. Such events, if widespread across the passerine phylogeny, could explain the fast changes in GRC genetic content between species (Kinsella et al. 2019; Schlebusch et al. 2023; Ruiz-Ruano et al. 2025). Future studies are needed to test whether occasional paternal inheritance is widespread beyond the zebra finch or not, and, if so, to quantify the paternal inheritance rate. Comparison of GRC and W-chromosome diversity and phylogeny could provide more insights into gene flux between GRC haplotypes and the relative importance of different selection pressures in explaining the observed extremely low genetic diversity in this chromosome. Our easy-to-use pipeline should be useful for the needed population genetic analyses of GRC of any size and can be easily extended to the study of sex chromosomes and other tissue-specific chromosomes.

In summary, we show that highly repetitive genomic regions such as the Australian zebra finch macro-GRC can be largely assembled with accurate long reads. We then provide an easy-to-use pipeline, ParaVar, to identify regions suitable for haplotype-based analysis along such a highly repetitive, tissue-specific chromosome, even using short-read libraries. We envision that ParaVar will be useful to perform population genetic analyses of GRCs of any size, and can be easily extended to the study of non-recombining sex chromosomes and some B chromosomes (e.g., Camacho 2005).

## Supplementary information

Supplementary materials

Supplementary tables

Data Set 1

## Data Availability

All sequencing data used in this work have been deposited in the Sequence Read Archive (HiFi and Omni-C data in PRJNA1419712, see accession number of previously published data in Table S2). The HiFi testis assembly and associated list of GRC contigs have been deposited in Figshare (https://doi.org/10.6084/m9.figshare.31073239). The key analysis files and scripts used to generate the main figures have been deposited in OSF (https://doi.org/10.17605/OSF.IO/YZ67D). The ParaVar pipeline implemented in this study is available on GitHub (https://github.com/ChenAugustin/ParaVar).
