# Supplementary material for: Paralog-aware assembly and filtering strategies reveal minimal nucleotide variation on the macro germline-restricted chromosome of the zebra finch
Source: Heredity (Edinb). 2026 Mar 16;135(8):571–81. doi: 10.1038/s41437-026-00830-z (PMC13545277; doi:10.1038/s41437-026-00830-z)
Supplement: Supplementary file 1 [file 41437_2026_830_MOESM1_ESM.docx]

# Supplementary materials for:

# Paralog-aware assembly and filtering strategies reveal minimal nucleotide variation on the macro germline-restricted chromosome of the zebra finch

# Supplementary figures

**Figure S1.** New GRC reference assembly (**A**) and experimental design (**B**). **A**: Previous between-population analyses of the zebra finch macro-GRC were only based on ~100 kb of GRC-linked sequence obtained via targeted read selection and assembly at testis-specific regions, which represents <1 % of the GRC size estimated at 167.3 Mb (Kinsella et al. 2019). Here, we take advantage of a new 90.39 Mb long-read GRC reference assembly, which contains 54.03 % of the estimated 167.3 Mb GRC size (Table S5), to conduct the first high-throughput between-population genetic study of the GRC specifically. **B**: GRC-linked nucleotide diversity was investigated across 9 matrilines from 6 different populations based on the mtDNA phylogeny of Pei et al. (2022a) to span the broadest range of nucleotide diversity available to us. For each individual, Illumina re-sequencing data were obtained from one somatic (liver or leg muscle) and one gonad (testis) tissue. We used the exact 9 individuals as in the analyses conducted by Pei et al. (2022a), allowing us to directly compare our results.

**Figure S2.** Density histogram of read depth per individual across the 5 high-confidence single-copy GRC-linked sequences from (Pei 2022b) with a full-length BLASTN hit in a GRC-linked contig of VGP+HiFi_GRC. Black lines indicate median (= 22) and red bins indicate sites with some level of somatic mismapping (SM > 0 %)

**Figure S3.** The effect of A-chromosomal mismapping on the shape of the trees was visualised using the SuperNetwork plugin from SplitsTree4 (Huson et al. 2004). No scale bar is provided for the SuperNetworks, as they are not expressed in substitutions per site but rather as “average relative length”, a relative unit that varies between SuperNetworks (Huson et al. 2004). SM = 0 % (s1), SM < 1 % (s2), SM < 10 % (s3), SM < 30 % (s4).

**Figure S4. Schematic representations of the three types of mismapping.** A target library containing reads from the control region (grey), the assembled target region (pink), and the unassembled target region (orange) is mapped against a reference genome containing the control and target regions, the latter being partially assembled. In the case of the GRC, mismapping of reads from A chromosomes onto GRC contigs (AChr-mm) is expected to add false positive GRC SNPs corresponding to differences between the A-chromosomal and GRC paralogs. Collapsing of reads from unassembled GRC regions onto their assembled counterparts (GRC-mm) is expected to add false-positive GRC SNPs corresponding to differences between assembled and unassembled GRC paralogs. Regions affected by GRC-mm will typically have a higher read depth proportional to the number of collapsed unassembled copies. Finally, genuine GRC SNPs along regions present in multiple and near-identical copies (dashed box) are expected to be lost due to ambiguous mapping of short reads (GRC-amb) as mappers typically assign reads randomly to one of their equally-likely targets.

**Figure S5. Gene density and completeness.** Gene density of all annotated genes across the 692 contigs of the concatenated GRC assembly, as shown in Figure 3A (top track). The second to fourth tracks show gene density based on DIAMOND (Buchfink et al. 2015) alignments of GRC-annotated proteins against the NCBI NR database, using match coverage thresholds of 100%, ≥90%, and ≥80%, respectively. The bottom track highlights the scorable GRC contigs identified in this study.

**Figure S6.** Nucleotide diversity on the scorable GRC across annotation features and for different filter thresholds on somatic mismapping (SM): 0 %, ≤ 1 %, ≤ 10 %, ≤ 30 %, which are labeled here s1, s2, s3, and s4, respectively.

**Figure S7.** Nucleotide diversity on scorable GRC regions annotated as coding sequence (CDS) comparing non-synonymous and synonymous SNPs, and for different filter thresholds on somatic mismapping (SM): 0 %, ≤ 1 %, ≤ 10 %, ≤ 30 %, which are labeled here s1, s2, s3, and s4, respectively.

**Figure S8.** Nucleotide diversity per 1-kb window across contigs with at least 1 scorable window when removing the filter on somatic mismapping. Shown in black are non-scorable windows across which π could not be calculated.

**Figure S9.** Nucleotide diversity per 1-kb window across contigs with at least 1 scorable window when keeping sites with somatic mismapping < 10 %. Shown in black are non-scorable windows across which π could not be calculated.

**Figure S10.** Only a few 1-kb windows have both a high scorability and high nucleotide diversity (π) on the GRC, especially when stringent filtering is applied against somatic mismapping (e.g., s1). Green vertical lines: 3 estimates of mitochondrial π (Figure 3A), black vertical lines: 5 estimates of A-chromosomal π (Figure 3A). Windows with high GRC-linked π are enriched in heterozygous genotypes, and some have a high somatic mismapping (s2, s3, s4) and are thus most likely mapping artifacts. Dot plot of GRC-linked nucleotide diversity (π) and scorability per 10-kb windows across different somatic mismapping (SM) thresholds: SM = 0 % (s1), SM < 1 % (s2), SM < 10 % (s3), SM < 30 % (s4). Note that scorability is at least 500/1-kb window as required by our filter on scorability. The scorability density distribution is also depicted to get a fuller picture.

**Figure S11**. Density distribution of statistics for mapping quality reveals a cluster with poor mapping quality and a cluster with good mapping quality. MQ is the mean mapping quality MAPQ per site and MQ0F is the fraction of reads with MAPQ 0.

**Figure S12.** Heterozygous GRC genotypes remain even when applying the most stringent filter on somatic mismapping (0% is required). This suggests that they are mismapping artifacts, which we confirmed via visual inspection.

**Figure S13.** Screenshot of alignment visualized on the Integrative Genomics Viewer (IGV). An example of variants we visually identified as artefacts caused by the mismapping of reads from unassembled GRC regions. The symptoms are clusters of read-phased variants with a heterozygous genotype called for almost all individuals, while we expect the GRC to be in a single copy (haploid) in males and hence yield only homozygous genotypes.

**Figure S14**. Screenshot of alignment visualized on the Integrative Genomics Viewer (IGV). Example of a variant we visually identified as an artefact caused by a copy number variant (CNV). The symptoms are reads supporting the alternative allele all present in a single individual with good mapping quality (MAPQ 60) and base quality (QV 41) forming a heterozygous site, while we expect the GRC to be in a single copy (haploid) in males and hence yield only homozygous genotypes.

**Figure S15**. Screenshot of alignment visualized on the Integrative Genomics Viewer (IGV). Example of variants we visually identified as genuine. The symptoms are reads from each individual supporting only one allele, either the reference or the alternative allele (homozygous genotypes), at each variant site, with polymorphism between individuals, and always good mapping quality (MAPQ 60).

# Supplementary data description

**Data S1.** Interactive dot plot alignment of our HiFi_tes assembly against the high-quality Australian zebra finch A-chromosomal reference genome from the Vertebrate Genome Project (VGP; GCA_003957565.4; Rhie et al. 2021).

# References

Buchfink B, Xie C, Huson DH (2015) Fast and sensitive protein alignment using DIAMOND. Nat Methods 12:59-60. <https://doi.org/10.1038/nmeth.3176>

Huson DH, Dezulian T, Klopper T, Steel MA (2004) Phylogenetic super-networks from partial trees. TCBB 1:151-158. <https://doi.org/10.1109/TCBB.2004.44>

Kinsella CM, Ruiz-Ruano FJ, Dion-Côté A-M, Charles AJ, Gossmann TI, Cabrero J et al. (2019) Programmed DNA elimination of germline development genes in songbirds. Nat Commun 10:5468. <https://doi.org/10.1038/s41467-019-13427-4>

Pei Y, Forstmeier W, Ruiz-Ruano FJ, Mueller JC, Cabrero J, Camacho JPM et al. (2022a) Occasional paternal inheritance of the germline-restricted chromosome in songbirds. PNAS 119:e2103960119. <https://doi.org/10.1073/pnas.2103960119>

Pei Y (2022b) Supporting information for “Occasional paternal inheritance of the germline-restricted chromosome in songbirds.” Open Science Framework. <https://doi.org/10.17605/OSF.IO/N9X2G>. Accessed 17 March 2022.

Rhie A, McCarthy SA, Fedrigo O, Damas J, Formenti G, Koren S et al. (2021) Towards complete and error-free genome assemblies of all vertebrate species. Nature 592:737-746. <https://doi.org/10.1038/s41586-021-03451-0>
