# Supplementary material for: Paralog-aware assembly and filtering strategies reveal minimal nucleotide variation on the macro germline-restricted chromosome of the zebra finch
Source: Heredity (Edinb). 2026 Mar 16;135(8):571–81. doi: 10.1038/s41437-026-00830-z (PMC13545277; doi:10.1038/s41437-026-00830-z)
Supplement: Supplementary file 2 [file 41437_2026_830_MOESM3_ESM.html]

D-GENIES - Dotplot large Genomes in an Interactive, Efficient and Simple way


Legend

Filter small matches:

Min identity (abs):

Strong precision

Stroke width:

Regions delimiters:

Loading...

How to cite
Cabanettes F, Klopp C. (2018) D-GENIES: dot plot large genomes in an interactive, efficient and simple way. PeerJ 6:e4958 https://doi.org/10.7717/peerj.4958

 


---

Copyright © 2020-2022 — INRAE | Copyright © 2017-2019 — INRA |
Designed by GenoToul Bioinfo platform.
